# Screening of immobilization method in aerogel matrix in the presence of protic ionic liquid

**DOI:** 10.1186/1753-6561-8-S4-P214

**Published:** 2014-10-01

**Authors:** Anderson Barbosa, Jessica Lisboa, Alini Fricks, Álvaro Lima, Elton Franceschi, Silvana Mattedi, Cleide Soares

**Affiliations:** 1 Instituto de Tecnologia e Pesquisa, Universidade Tiradentes, Aracaju, SE, Brazil; 2 Núcleo de Estudos em Sistemas Coloidais, Universidade Tiradentes, Aracaju, SE, Brazil; 3 Universidade Federal da Bahia, Salvador, Bahia, BA, Brazil

## 

Aerogel and xerogel are formed via hydrolysis and polycondensation reactions of silica precursors, such as tetraethylorthosilicate (TEOS), always careful not to cause collapse, reduction in surface area and pore size. Several studies shown the use sol-gel entrapment possesses a number of desirable attributes, as the enzyme is physically entrapped in a rigid glass framework that permitted stabilization of the enzymes, terciary structure owing to the tight gel network. In this study, have focused on the screening of immobilization method in aerogel matrix in the presence of protic ionic liquid (PIL) with evaluation catalytic efficiency and operational stability. The novel mesoporous silica supports (aerogels) obtained by the sol-gel technique was used to immobilize commercial *Burkholderia cepacia *(BC) lipase by physical adsorption, covalent binding and encapsulation in the absence and presence de protic ionic liquid (N-methylmonoethanolamine pentanoate - C_5_). Catalytic efficiency was determined in the analyses of hydrolytic activities were carried out on the lipase loading solution and immobilized enzyme to determine the total activity recovery yield, Ya (%). For physical adsorption the recovery of activity with PIL was 82.95% (ADSLI) and in the absence 70.31% (ADS). Operational stability of the enzyme has also been examined and obtained similar values a half-life of 0.73 h to ADSLI and 0.88 h for ADS occurs with 2 batches. And other immobilized technique, covalent binding the results showed that in aerogels supports in the presence of C_5 _obtained 69.13% (CBLI) of total activity recovery yield and 39.11% (CB). Under such conditions, the operational stability tests indicated that a small enzyme deactivation occurs after 15 batches, revealing a biocatalyst half-life of 7.5 h for CBLI. For encapsulation, the recovery of activity with PIL (ENLI) was 45% and 37% in the absence PIL (EN). ENLI exhibited a half life of more than 23 batches with 70% of its activity remaining, with around 11.5 h. And EN the half-life was 6.5 hours, totaling 13 batches. Therefore, ADSLI presented good efficiency with total activity recovery yield but for operational stability ENLI showed the best between immobilization methods studied. Hence, it is rather appropriate for application in hydrolytic processes.

